# Pulmonary tuberculosis and multidrug-resistant *Mycobacterium tuberculosis* in northwestern Ethiopia: a hospital-based cross-sectional study among presumptive pulmonary tuberculosis patients

**DOI:** 10.3389/fmed.2023.1266780

**Published:** 2023-12-12

**Authors:** Birhanu Wubu, Mohabaw Jemal, Yihenew Million, Mucheye Gizachew

**Affiliations:** ^1^Department of Clinical Laboratory, Abrihajira Hospital, Amhara National Regional State, Abrihajira, Ethiopia; ^2^Department of Medical Microbiology, School of Biomedical and Laboratory Sciences, College of Medicine and Health Sciences, University of Gondar, Gondar, Ethiopia

**Keywords:** Abrihajira and Metema hospital, Xpert MTB/RIF assay, multidrug-resistant *Mycobacterium tuberculosis*, line probe assay, northwestern Ethiopia

## Abstract

**Introduction:**

Border areas are important sites for disseminating *Mycobacterium tuberculosis* among individuals living in such areas. This study examined patients with suspected pulmonary tuberculosis (PTB) visiting the Abrihajira and Metema hospitals in northwest Ethiopia to investigate the prevalence of rifampicin-resistant *Mycobacterium tuberculosis* (RR-MTB), multidrug-resistant *Mycobacterium tuberculosis* (MDR-MTB), and risk factors related to *Mycobacterium tuberculosis* infection.

**Methods:**

A hospital-based cross-sectional study was conducted from February to August 2021 among 314 PTB presumptive patients. Xpert MTB/RIF and line probe assays (LPA) were used to process sputum samples. Data were imported into the Epi-Data 3.1 program and exported to Statistical Package for the Social Sciences (SPSS) version 20.0 (SPSS, Chicago, IL, United States) to conduct the analysis. A logistic regression analysis was used to investigate the relationship between the dependent and independent variables. A value of *p* of <0.05 denoted statistical significance.

**Results:**

Of the total (314) PTB presumptive patients who participated in this study, 178 (56.69%) were men, and 165 (52.5%) were from 25 to 50 years of age with a median age of 35.00 (inter-quartile: 25–45 years). Among all patients, 12.7% had PTB by Gene Xpert and 7/314 (2.23%) were resistant to rifampicin. Among patients enrolled, 4/314 (1.27%) had MDR-MTB (resistant to RIF and INH) by LPA. Regarding the risk factors assessed, primary level of education, sputum production, night sweating, respiratory disorder, contact history of TB, history of MDR-MTB infection, history of alcohol use, and cigarette smoking showed statistical significance with the prevalence of PTB (*p ≤ 0.05*).

**Discussion:**

This study observed a high prevalence of PTB, RR-MTB, and MDR-MTB compared with many other previous studies conducted in Ethiopia. Among the assessed risk factors that could be associated with the prevalence of PTB, eight were statistically significant. This prevalence, resistance, and statistically significant variables are the evidence to which more emphasis should be given to the country’s border areas.

## Introduction

Pulmonary tuberculosis (PTB) is a disease owing to *M. tuberculosis* that affects predominantly the lungs (1). It is the world’s number one killer among infectious diseases and one of the top 10 causes of death (1, 2). Multidrug-resistant *Mycobacterium tuberculosis* (MDR-MTB) is the most important global public health issue, and a study depicted that biocide affects multiple sites in the bacterium and found that it can cause resistance in a non-specific way (3). In 2018, there were close to 10 million people with tuberculosis and 1.2 million tuberculosis-related deaths among human immunodeficiency virus (HIV)-negative people globally. Approximately 500,000 new cases of rifampicin-resistant (RR) tuberculosis also occurred in the same year, 70% of which were classified as multidrug-resistant tuberculosis (MDR-TB) (1, 4). The high prevalence of HIV, being in border regions, unstable residency, delayed early diagnosis, and inadequate early treatment initiation all contribute to the high burden of tuberculosis (TB) and MDR-TB in low-income nations (5). A disease management and control program must implement quick identification, early treatment initiation, ongoing surveillance, and regular monitoring of drug-resistant TB (6).

In December 2010, the World Health Organization (WHO) approved Xpert MTB/RIF (Cepheid, Sunnyvale, CA, USA). This automated molecular system detects both MTB and rifampicin-resistant (RR) tuberculosis concurrently to address the issue of early TB diagnosis in low-income countries (7). In over 90% of cases, RR-MTB is a surrogate marker for MDR-TB. It is primarily recommended for patients with TB/HIV co-infection, suspected MDR-TB, and pediatric patients, though it was indicated for all TB presumptive patients as of 2013 (8, 9). To diagnose TB and RR-MTB, Ethiopia introduced the Xpert MTB/RIF assay in 2014 (10). Ethiopia ranks 15^th^ among high MDR-TB countries, with more than 5,800 projected MDR-TB cases yearly, making it one of the 30 countries with the highest TB, TB/HIV, and MDR-TB burdens (11).

Only a few Xpert MTB/RIF test investigations have been performed in Ethiopia. For example, Ataye (8.98%) (12), Eastern Amhara, Ethiopia (11%) (13), and Tigray (7.9%) reported a prevalence of 7.9–11% and 5.3–9% for MTB and RR-MTB, respectively. Only a few investigations on presumed PTB patients were undertaken in Ethiopia until 2021 in the nation’s border regions. These studies do not adequately reflect the PTB status of the entire country’s border region. Therefore, having representative data in the nation’s border region will close the knowledge gap and give policymakers and implementers up-to-date information.

## Materials and methods

### Study design, period, and area

A hospital-based cross-sectional study was conducted from February to August 2021 at Metema and Abrihajira hospitals. These hospitals are located in the West Gondar region of the Amhara National Regional State, 850 km from the capital of Ethiopia, Addis Ababa. These two hospitals offer a range of patient services, including outpatient, inpatient, gynecological, tuberculosis, and antiretroviral therapy (ART) clinics and various diagnostic and treatment services for other illnesses in the community. There are around 119 beds in the two hospitals: 85 beds at the Metema Hospital and 34 beds at the Abrihajira Hospital. It borders Eritrea to the north and Sudan to the west, and large-scale agricultural activities are being practiced in these areas of Ethiopia (14, 15).

### Source and study population

The source population was all patients who were visiting Metema and Abrihajira hospitals. The study population was all the PTB presumptive patients seeking health services at the specified hospitals.

### Sample size determination

The sample size was calculated using a single population proportion formula and a 5% margin of error at a 95% confidence interval (CI). The target populations for the study were all PTB presumptive patients at the Metema and Abrihajira hospitals, which served an estimated 160,000 people (*N* value) (13, 16). The prevalence (p) of PTB obtained from the previous study conducted at the University of Gondar Hospital, Northwest Ethiopia, was 24.6% (17).

n = $Z^{2}$ p(1-p) = ( $1.96){}^{2}$ (0.246) (0.754) = 285


$$d^{2}\;\;0.0025$$

Where Z = 95% confidence interval (1.96);

p = prevalence rate from the previous study;

d = Margin of sampling error (5%);

q = 1-p;

*n* = number of sample units that will be studied.

With a 10% non-respondent rate in mind, 285 + 29 = 314 patients comprised the entire sample used in the current study. All of these study participants were included using a convenience sampling technique.

### Socio-demographic data collection

Socio-demographic data were collected using a structured, closed-ended questionnaire-based face-to-face interview. A questionnaire covering the social-demographic, clinical, and PTB-related risk factors was formulated in English, then translated into the local (Amharic) language for data collection and retranslated into English for analysis and reporting.

### Specimen collection, handling, and transportation

Based on the national TB diagnosis guideline (18), spot or morning sputum specimens were collected using two falcon tubes with 4–5 mL from each PTB presumptive patient. The first falcon tube sputum was stored at 2–8°C for a maximum of 1 week until testing at the Abrihajira and Metema primary hospitals, and the second one was stored at −20°C and transported to the University of Gondar Comprehensive Specialized Hospital by triple package systems at 2–4°C using an ice pack for solid culture (LJ) when the first specimen was positive for MTB/RIF by Gene Xpert MTB/RIF nested real-time polymerase chain reaction (PCR) at the Abrihajira and Metema hospitals and MDR-MTB testing by line probe assays (LPA) at the Amhara Public Health Institute.

The MTB/RIF Xpert Assay was conducted using the Gene Xpert machine model number 900-0513GxIvR2/Rev.C.2 (Cepheid, Inc.) to detect PTB and rifampicin (RIF) resistance, as described in previous studies (19, 20). Decontaminated samples were mixed with sodium hydroxide (NaOH) and isopropanol sample reagent buffer (1:3 ratio) and incubated at room temperature for 15 min. The GeneXpert MTB/RIF (Cepheid, Sunnyvale, Inc.) was loaded with 2 milliliters (mL) of the sample in the cartridge (GeneXpert MTB/RIF, Cepheid, Sunnyvale, CA). Results were generated after 2 h, indicating whether *Mycobacterium tuberculosis* was present and its RIF sensitivity.

Seven specimens positive for RR-MTB were inoculated onto Lowenstein–Jensen (LJ) slants, following previous methods (20, 21). They were incubated at 37°C for 4–6 weeks and colonies were checked twice weekly. LJ slant medium was considered positive when colonies appeared, confirmed by AFB smears.

After GeneXpert MTB/RIF (Cepheid, Sunnyvale, CA) examination, LPA was performed at the Amhara Public Health Institute Laboratory following the manufacturer’s protocol (Hain Life Science GmbH, Nehren, Germany) to detect RIF and Isoniazid (INH) resistance due to mutations in *rpo, inhA*, and *katG* genes. The hybridization procedure included denaturation, conjugation, and substrate application. As in a previous study, results were interpreted for *Mycobacterium tuberculosis* presence, resistance, sensitivity, or invalidity (18).

### Operational definitions

Presumptive PTB patients: Patients were considered to have ‘presumptive TB’ if any of the following symptoms, regardless of duration, were present: cough, fever, night sweats, unintentional weight loss, chest pain, or loss of appetite.

Confirmed PTB: TB confirmed through bacteriological testing, with the detection of TB in a sputum sample using Xpert® MTB/RIF, was considered confirmed TB (22).

### Quality control

The study used a pretested questionnaire in a comparable environment that was not part of the study. Close supervision and support during data collection and daily checks of completed questionnaires were made for correctness, completeness, and clarity of data. Internal and external quality checks were performed for the Gene Xpert assay AFB. Pre-analytical, analytical, and post-analytical quality control methods were used to ensure the reliability and validity of the study test procedures for sample collection, processing, and evaluation. A recently sub-cultured *M. tuberculosis* H37Rv strain was suspended for culture and LPA quality control. The biosafety cabinet is where all the culture’s handling and collection materials, including used pipettes and tubes, were kept. All materials were sealed before being placed in the autoclave, and gloves and other garbage were gathered in a plastic bag for the autoclave.

### Data analysis

To conduct the analysis, the obtained data were input into the “Epi-Data 3.1” program and exported to Statistical Package for the Social Sciences (SPSS) version 20.0 (SPSS, Chicago, IL, USA). The data were compiled using descriptive statistics. The link between the dependent-and independent-variables (clinical features, socio-demographic characteristics, and predisposing factors) was investigated using bivariate and multivariate analysis. For the multivariable analysis, variables that demonstrated significance at *p*-values of 0.2 in the bivariate analysis were chosen. As measures of the strength of association, adjusted odds ratios (AOR) and their 95% confidence intervals (CIs) were used. Statistical significance was defined as a value of *p* ≤0.05.

## Results

### Socio-demographic data

The study included 314 PTB presumptive patients, whose median age was 35.0 (inter-quartile: 25–45 years), and those treated at the Metema and Abrihajira hospitals. Men comprised the majority of research participants (178; 56.69%), followed by married people (170; 54.1%), those who could not read or write (121; 38.5%), people who lived in cities (162; 51.6%), and people aged 25 to 50 years (165; 52.5%) (Table 1).

**Table 1:** Socio-demographic characteristics of PTB presumptive patients in northwestern Ethiopia, 2021 (*N* = 314).

Variables	Categories of variables	Frequency (*n*)	Percent (%)
Age	<25	86	27.4%
	25–50	165	52.5%
	51–75	58	18.5%
	>75	5	1.6%
Sex	Male	178	56.69%
	Female	136	43.31%
Residence	Urban	162	51.6%
	Rural	152	48.4%
Marital status	Unmarried	95	45.9%
	Married	170	54.1%
Educational status	Unable to read and write	121	38.5%
	primary level	88	28.0%
	Above primary level	105	33.4%
Occupation	Governmental	88	28%
	Private	64	20.4%
	Housewife	41	13%
	Labor worker	32	10.2%
	Farmer	93	29.6%
Monthly income Ethiopian birr	<1500.00	91	29.0%
	1500.00–3000.00	93	29.6%
	3000.00–4700.00	55	17.5%
	>4700.00	75	23.9%

### Clinical presentation and behavioral data

All the study individuals included in this investigation displayed PTB clinical signs and symptoms, as illustrated in Table 2. Cases with loss of appetite (280, 89.2%), sputum production (276, 87.89%), weight loss (241, 76.75%), and night sweats (239, 76%) were the participants’ primary symptoms. Of all study participants, 94 (29.9%) had past contact with patients who were known to be infected with TB, 29 (9.2%) had previous contact with patients who were known to have MDR-TB, 62 (19.7%) had a history of alcohol use, 29 (6.9%) had a history of PTB, and 30 (9.6%) had a history of smoking cigarettes (Table 2).

**Table 2:** PTB among PTB presumptive patients in relation to their clinical presentations in northwestern Ethiopia, 2021 (*N* = 314).

Variables		PTB status		Total no. (%)
		Positive	Negative	
Weight loss	Yes	35 (14.5%)	206 (85.5%)	241 (76.75%)
	No	5 (7%)	68 (93%)	73 (23.25%)
Night sweating	Yes	37 (15.5%)	202 (84.5%)	239 (76%)
	No	3 (4%)	72 (96%)	75 (24%)
Sputum production	Yes	39 (14%)	237 (86%)	276 (87.89%)
	No	1 (2.6%)	37 (97.4%)	38 (12.11%)
Fever	Yes	37 (14%)	228 (86%)	265 (84%)
	No	3 (6%)	46 (94%)	49 (16%)
Respiratory disorder	Yes	22 (19%)	94 (81%)	116 (36.94%)
	No	18 (9%)	180 (91%)	198 (63.06%)
Loss of food appetite	Yes	39 (14%)	241 (86%)	280 (89.2%)
	No	1 (3%)	33 (97%)	34 (10.8%)
Chest pain	Yes	39 (14.2%)	236 (85.8%)	275 (87.6%)
	No	1 (2.6%)	38 (97.4%)	39 (12.4%)
TB family history	Yes	18 (19.15%)	76 (80.85%)	94 (29.9%)
	No	22 (10%)	198 (90%)	220 (70%)
Smoking history	Yes	10 (33.33%)	20 (66.67%)	30 (9.6%)
	No	30 (10.56%)	254 (89.44%)	284 (90.4%)
Alcohol use history	Yes	16 (25.8%)	46 (74.2%)	62 (19.7%)
	No	24 (9.5)	228 (90.5%)	252 (80.3%)
Contact history with MDR-TB patients	Yes	10 (34.48%)	19 (65.52%)	29 (9.2%)
	No	30 (10.52%)	255 (89.48%)	285 (90.8%)

### Prevalence of PTB, RR-MTB, and MDR-MTB

Forty (12.7%) of the study’s subjects were PTB-positive. The prevalence of RR-MTB was 2.2% across the entire study population (7/314) and was 17.5% (95% CI: 9.1–25.9) among individuals with PTB confirmation. MDR-MTB cases were 10% of the PTB confirmed cases and four (1.27%) of the study’s total participants. Study participants who were male, married, had only completed their primary education, were private workers, and had lower monthly incomes were shown to have higher PTB. Additionally, more men with RR-MTB and MDR-MTB were seen in the 25 to 50 age group among rural residents and unmarried research participants (Table 3).

**Table 3:** PTB, RR-MTB, and MDR-MTB among PTB presumptive patients in northwestern Ethiopia, 2021 (*N* = 314).

Variables		Participants number	PTB positive, *n* (%)	RR-MTB positive, *n* (%)	MDR-MTB positive, *n* (%)
Age	<25	86	12 (13.9%)	1 (1.2%)	1 (1.2%)
	25–50	165	24 (14.6%)	6 (3.6%)	3 (4.6%)
	51–75	58	4 (10.3%)	0 (0%)	0 (0%)
	>75	5	0 (0%)	0 (0%)	0 (0%)
Sex	Male	178	25 (14%)	6 (3.4%)	4 (2.3%)
	Female	136	15 (11%)	1 (0.7%)	0 (0%)
Residence	Urban	162	21 (13%)	2 (1.2%)	1 (0.6%)
	Rural	152	19 (12.5%)	5 (3.3%)	3 (2%)
Marital status	Married	95	16 (16.8%)	3 (3.2%)	1 (1.1%)
	Unmarried	170	24 (14%)	4 (2.4%)	3 (1.8%)
Educational status	unable to read & write	121	15 (12.4%)	2 (1.7%)	1 (0.8%)
	primary level	88	17 (19.3%)	2 (2.3%)	1 (1.1%)
	Above primary level	105	8 (7.6%)	3 (2.9%)	2 (2%)
Occupation	Governmental	64	3 (4.7%)	0 (0%)	0 (0%)
	Private	41	11 (26.8%)	1 (2.4%)	1 (2.4%)
	House wife	32	3 (9.4%)	0 (0%)	0 (0%)
	Labor worker	93	11 (11.8%)	3 (3.2%)	2 (2.2%)
	Farmer	88	12 (13.6%)	3 (3.4%)	1 (1.1%)
Monthly income ETB*	<1500.00	91	15 (16.5%)	3 (3.3%)	2 (2.2%)
	1500.00–3000.00	93	12 (12.9%)	2 (2.2%)	1 (1.1%)
	3000.00–4700.00	55	6 (10.9%)	1 (1.8%)	0 (0%)
	>4700.00	75	7 (9.3%)	1 (1.3%)	1 (1.3%)

*-Ethiopian Birr, N = number, PTB-pulmonary tuberculosis, RR-MTB-rifampicin resistance-M. tuberculosis, MDR-MB-multidrug resistance-M. tuberculosis; ETB-Ethiopian Birr.

### Clinical risk factors associated with PTB

The majority of the subjects showed typical clinical signs and symptoms, which included nocturnal sweating (239, 76.1%), weight loss (241, 76.8%), coughing up sputum (274, 87.3%), and loss of appetite (280, 89.2%). The bi-variable analysis of patient characteristics revealed that patients’ lack of appetite, sputum production, night sweats, respiratory pain, fever, weight loss, and chest pain were significant clinical aspects. However, the multivariable model indicated that night sweating (AOR = 4.24 CI; 1.14–15.78), respiratory problems (AOR = 2.08 CI; 1.04–4.16), and sputum production (AOR =22.62 CI; 1.24–411.86) were significantly associated with PTB (Table 4).

**Table 4:** Association of the clinical variables with PTB among PTB presumptive patients in northwestern Ethiopia, 2021 (*N* = 314).

Variables		PTB status		COR (95%CI)	*p*-value	AOR (95%CI)	*p*-value
		Positive	Negative				
Weight loss	Yes	35 (14.5%)	206 (85.5%)	4.4 (1.3–14.7)	0.016	0.84 (0.26–2.7)	0.765
	No	5 (7%)	68 (93%)	1		1	
Night sweating	Yes	37 (15.5%)	202 (84.5%)	2.3(0.87–6.13)	0.093	4.24 (1.1–15.8)	0.031*
	No	3 (4%)	72 (96%)	1		1	
Sputum production	Yes	39 (14%)	237 (86%)	6 (0.81–45.7)	0.079	23 (1.2–41.9)	0.035*
	No	1 (2.6%)	37 (97.4%)	1		1	
Fever	Yes	37 (14%)	228 (86%)	2.5 (0.74–8.4)	0.143	2 (0.197–19.6)	0.564
	No	3 (6%)	46 (94%)	1			
Respiratory disorder	Yes	22 (19%)	94 (81%)	2 (1.20–4.6)	0.013	2.1 (1.04–4.2)	0.037*
	No	18 (9%)	180 (91%)	1		1	
Loss of food appetite	Yes	39 (14%)	241 (86%)	5 (0.71–40.2)	0.104	0.96(0.01–68)	0.984
	No	1 (3%)	33 (97%)	1		1	
Chest pain	Yes	39 (14.2%)	236 (85.8%)	6.28 (0.84–47)	0.074	3 (0.35–24.5)	0.326
	No	1 (2.6%)	38 (97.4%)	1		1	

Only significant values (*P*-value) are indicated by an asterisk indicator (*); 1 = Indicates the reference group.

### Factors associated with PTB among PTB presumptive patients

Ten (34.48%) patients had a contact history with MDR-TB patients, 10 (33.33%) had a history of smoking cigarettes, and 18 (19.15%) of the study participants had a contact history with known PTB-infected patients. Patients with primary education (AOR = 3.76 CI; 1.3–10.5), contact history with confirmed PTB cases (AOR = 2.06 CI; 1.00–4.22), a history of alcohol use (AOR = 2.93 CI; 1.40–6.10), and a history of smoking cigarettes (AOR = 3.18 CI; 1.298–7.77) were more likely to contract the PTB infection than were patients without these risk factors (Table 5).

**Table 5:** Association of the demographic and behavioral characteristics with PTB among PTB presumptive patients in northwestern Ethiopia, 2021 (N = 314).

Variables	Categories	PTB status		COR (95%CI)	*p*-value	AOR (95%CI)	*p*-value
		Positive	Negative				
Educational level	Unable to read and write	15 (12.4%)	106 (87.6%)	0.8 (1.1–3.03)	0.240	2(0.7–5.98)	0.18
	primary level	17 (19.3%)	71 (80.7%)	1.1(0.01–7)	0.020	3.8 (1–11)	0.012*
	Above primary level	8 (7.6%)	97 (92.4%)	1		1	
TB family history	Yes	18 (19.15%)	76 (80.85%)	2.36 (1.2–4.6)	0.013	2.1 (1.01–4.2)	0.049*
	No	22 (10%)	198 (90%)	1		1	
Smoking history	Yes	10 (33.33%)	20 (66.67%)	4.23 (1.8–9.9)	0.001	3.2 (1.3–7.77)	0.011*
	No	30 (10.56%)	254 (89.44%)	1		1	
History of alcohol use	Yes	16 (25.8%)	46 (74.2%)	3.30 (1.6–6.7)	0.001	2.9 (1.4–6.10)	0.004*
	No	24 (9.5)	228 (90.5%)	1		1	
Contact history with MDR-TB cases	Yes	10 (34.48%)	19 (65.52%)	4.47 (2–10.5)	0.001	3.9 (1.59–9.5)	0.003*
	No	30 (10.52%)	255 (89.48%)	1		1	

Only significant values (*P*-value) are indicated by an asterisk indicator (*); 1 = Indicates the reference group.

## Discussion

The prevalence of pulmonary tuberculosis (PTB) in the current study was 12.7% (95% confidence interval: 9.2–16.6). This figure aligns with the findings from other studies conducted in similar settings, such as Addis Ababa Saint Paul’s Hospital, Ethiopia (10%) (23), Northeast Ethiopia (13.5%) (24), South Africa (11.79%) (25), and Nigeria (12.3%) (26). However, it is worth noting that our study reports a lower prevalence compared to research conducted in Debre Markos (19.8%) (27) and Gambella (20.0%) (28) in Ethiopia, as well as in China (32.12%) (29).

It is important to emphasize that the prevalence of PTB in our study is higher among PTB presumptive patients compared to some previous investigations within Ethiopia, such as Ataye, northeast Ethiopia (8.98%) (12), and Tigray, northwest Ethiopia (7.9%) (16). The variations in PTB prevalence observed across these studies may be attributed to differences in the characteristics of the study populations, geographical locations, and variations in laboratory techniques employed. Furthermore, it is noteworthy that the influx of daily laborers with low socioeconomic status from various regions of the country to the Metema and Abrihajira areas, where extensive agricultural activities take place, and the proximity of these study areas to the border with Sudan and Eritrea, might contribute to a lower level of awareness regarding the risk of PTB transmission from individuals with a history of TB (30, 31).

The risk of contracting pulmonary tuberculosis (PTB) is amplified among individuals of disadvantaged socioeconomic status, experiencing compromised immune function, engaging in smoking habits, and having proximity to actively ill PTB patients (12). The current study indicated that participants with a primary level of education faced approximately a 4-fold increased susceptibility to PTB compared to individuals with higher levels of educational attainment (10). Notably, individuals with lower educational attainment or limited literacy may exhibit deficiencies in fundamental health literacy, potentially hindering their understanding of PTB transmission and associated risk factors. Extensive global research corroborates these suppositions (32, 33).

According to this study, PTB presumptive patients with frequent interaction with confirmed PTB patients were 2.1 times more likely to have the illness than those without such contacts. Similarly, research in Ethiopia revealed a high correlation between PTB and previous interactions with PTB patients (32, 34). A significant risk factor for developing PTB was previous interaction with MDR-TB subjects. In comparison to individuals who did not have such a background, suspected PTB patients who had frequent interaction with MDR-TB patients were nearly four times more likely to develop the PTB. Another study in Ethiopia revealed a statistically significant correlation between PTB prevalence among PTB presumptive patients and interaction with MDR-TB patients (35). The current study identified cigarette smoking as a risk factor for PTB, and those who smoked had a 3-fold higher risk of developing the condition than non-smokers. In the current study, patients with a history of alcohol use were also nearly three times more likely than those who did not acquire PTB illness. Numerous studies have shown that drinking alcohol and smoking cigarettes directly harm blood vessels and biliary function, lowering immunity and increasing the risk of PTB (36–39).

In the present study, individuals manifesting symptoms of night sweats, respiratory distress, or sputum production exhibited a respective 4.2-fold, 2-fold, and 23-fold increased likelihood of having pulmonary tuberculosis (PTB) compared to their counterparts. This observation finds support in a prior study, which established a statistically significant association between PTB and the presence of sputum production and nocturnal sweating (32).

In contrast to the current study, research conducted at Addis Ababa and Ataye Primary Hospital in Ethiopia revealed no statistically significant correlations between PTB and the symptoms of night sweats, respiratory difficulties, or sputum production (10, 38). The disparities observed in these results may be attributed to variances in the study populations and geographical settings.

Gene Xpert assay-identified RR-MTB isolates are indicators of MDR-MTB. The presence of RR-MTB poses a significant global health (12). Rifampicin-resistant MTB is significantly prevalent in low-income countries, which may be explained by their socioeconomic and demographic makeup, including their high rates of malnutrition, overcrowding, inadequate medical treatment, and lack of social security (12). In the present study, the prevalence of rifampicin-resistant *Mycobacterium tuberculosis* (RR-MTB) was 7 out of 314 participants (2.2%) across the entire cohort. Among the subset of PTB-confirmed patients, this prevalence was notably higher at 7 out of 40 (17.5%). This finding closely aligns with prior research conducted on confirmed PTB patients in Gondar, Ethiopia, which reported a prevalence of 15.8% [17], as well as studies in Nigeria (13.9%) (40) and North India (10.5%) (41). However, it should be noted that our study observed a higher RR-MTB prevalence than previous investigations among confirmed PTB patients in Ataye (5.3%) (12), Afar (4.3%) (42), and Gambella (4.9%) (17), all within the Ethiopian context. This disparity can be elucidated by several factors within the study population, including a limited understanding of the mechanisms leading to drug resistance, restricted access to healthcare facilities, potential inaccuracies in patient diagnosis, treatment, and post-treatment monitoring, as well as suboptimal patient adherence. These factors may collectively account for the increased prevalence of rifampicin-resistant *Mycobacterium tuberculosis* (RR-MTB) observed in the present study [28]. However, the prevalence of RR-MTB is less common (33.3%) than that of the previous study conducted in Oromia, Ethiopia (43). We included suspicious individuals to identify PTB, whereas the study of the Oromia region included confirmed cases, which may have contributed to this variation. The discrepancies in prevalence across studies may also be explained by the diagnosis method, population geography, and study sets. The disparity in RR-MTB across the country may also be attributed to variations in patient selection and the study’s sample size. In the current investigation, the prevalence of MDR-MTB was 4/314 (1.27%) across all patients and 4/40 (10%) among PTB-confirmed individuals. This result is comparable to investigations done in Ethiopia among confirmed patients, where the rates were 7.8% (44) and 13.8% (45), respectively. The current study’s findings were less prevalent than those in Tigray, Ethiopia (16.7%) (45). We included presumptive cases, but other studies included recognized patients of RR-MTB to verify drug resistance profiles, which may have contributed to this variation. The results of the current study, however, were more significant than those of investigations conducted in South Gondar, Ethiopia (1.8%) (46), Central Ethiopia (1.5%) (47), and India (1.34%) (48). The disparities in access to medical services and health education and the presence of a considerable labor force and a transient resident population at the study site, as opposed to other regions, could plausibly account for the observed variation, as highlighted in this study.

## Conclusion

After attempting to compare the results of the current study with numerous other prior studies conducted in Ethiopia, we found a greater prevalence of PTB, RR-MTB, and MDR-MTB. Five risk factors were statistically significant (*p*≤ 0.05) when considered as having a potential relationship with PTB prevalence. The high incidence, resistance, and statistically significant variables found in this study support the idea that national border regions should receive more attention. The findings of this study in the border region will fill knowledge gaps and give researchers, decision-makers, and implementers up-to-date information. The results should also disturb the scientific community because an additional study is required if only to lessen the total impact of tuberculosis in the region.

## Data availability statement

The original contributions presented in the study are included in the article/supplementary material, further inquiries can be directed to the corresponding author.

## Ethics statement

Ethical approval (SBMLS/2724/2021) was obtained from the research and ethics review committee of the School of Biomedical Laboratory Sciences, College of Medicine and Health Sciences, University of Gondar. The studies were conducted in accordance with the local legislation and institutional requirements. Written informed consent for participation in this study was provided by the participants' legal guardians/next of kin.

## Author contributions

MG: Conceptualization, Software, Supervision, Validation, Visualization, Writing – original draft, Writing – review & editing. YM: Software, Supervision, Validation, Visualization, Writing – review & editing. MJ: Software, Supervision, Validation, Visualization, Writing – review & editing. BW: Conceptualization, Data curation, Formal analysis, Writing – original draft.
